# Breastfeeding rates in Israel and their health policy implications

**DOI:** 10.1186/s13584-025-00689-1

**Published:** 2025-05-13

**Authors:** Deena R. Zimmerman, Nati Brooks, Janice Wasser, Linoy Vaknin-Alon, Tunie Dweck, Sharon Alroy-Preis

**Affiliations:** 1 Public Health Directorate, Ministry of Health, Jerusalem, Israel; 2 Digital and Data Technologies Division, Ministry of Health, Jerusalem, Israel

**Keywords:** Exclusive breastfeeding, Demographics, National health policy, Trends

## Abstract

**Background:**

Monitoring breastfeeding rates has important health policy implications, as breastfeeding has significant positive impacts on maternal and child health and healthcare costs. This up-to-date, national, population-based breastfeeding rates study in Israel provides important information for health policy development.

**Methods:**

Breastfeeding rates were determined for the years 2016–2022 by retrospective analysis of Machshava Briah electronic medical records used by many Israeli Maternal and Child Health Clinics. This reflects approximately 70% of Israeli children with a nationwide distribution. Comparisons were conducted measuring breastfeeding rates over time and between different sub-groups.

**Results:**

The dataset consists of 945,437 infant records. The percentages of women with any breastfeeding as well as exclusive breastfeeding have shown a gradual decline annually from 2016 to 2022 and are lower than international goals. Sub-group analyses were conducted for 2022. Breastfeeding rates were higher among multipara mothers (versus primapara). Singleton mothers had much higher breastfeeding rates than twin mothers with the difference even more pronounced in exclusive breastfeeding rates. Mothers of preterm infants (< 37 weeks) and low birthweight infants breastfed less than mothers of full term infants and normal birthweight and were less likely to exclusively breastfeed. Mothers living in urban areas had the highest rates of breastfeeding and those living in rural areas had the lowest.

A subanalysis performed at two months postpartum for 2022 found the effect of maternal age with the highest rates of breastfeeding among 20–24 year old mothers. Inter-pregnancy interval also had an effect with the highest rates among those whose last pregnancy was 21–33 months ago and the lowest rates among those with an interval of < 1 year.

**Conclusions:**

The population-based data provides an important baseline marker. This study shows a drop in breastfeeding rates, indicating a need to investigate reasons for discontinuing breastfeeding and identifying possible areas for offering support. This data and similar follow-up studies provide the background evidence to warrant that Ministry of Health policies in the hospitals and in the community, help accomplish their goals.

**Supplementary Information:**

The online version contains supplementary material available at 10.1186/s13584-025-00689-1.

## Background

Breastfeeding, exclusively for six months and combined with complimentary feeds until the age of two years, is internationally recommended as the norm for infant nutrition [1, 2]. This practice has been associated with decreased risk of many pediatric and maternal diseases, both acute and chronic [3, 4]. Therefore, as part of public health planning, it is important for countries to monitor breastfeeding rates.

Until 2016, there were no annual statistics of breastfeeding available in Israel. Until that point, most studies were carried out on a portion of the population, divided by either geographic area or ethnic background. Questions regarding breastfeeding were included in a number of national surveys. For 2009–2012, a national nutrition survey was based on a cohort of 2000 infants who were followed up to two years of age [5]. This study showed that 90.7% of women intended to breastfeed with 20.0% exclusively breastfeeding at six months. The latest survey, which includes 2019–2020 data, shows that 90.1% of mothers intended to breastfeed [6]. The rate for any breastfeeding at six months was 35.6% (exclusive breastfeeding was 15.3%). There was an increase between the first and second survey for attempts to breastfeed in the first hour after birth (from 41.4 to 62.3%).

In Israel, pediatric preventive health care (well-child care) is provided uniformly to all children from birth to age six years, free of charge in Maternal and Child Health Clinics (MCHCs) by public health nurses and physicians [7]. Each of the 11 scheduled visits from one week to 6 years of age follows a clear format including routine immunizations, determining parental concerns, measuring and plotting the child’s growth, structured developmental assessment and anticipatory guidance regarding nutrition, child development and family dynamics. Information from the visit, including the infant’s feeding practices, is documented using an electronic medical record (EMR).

## Methods

Aim: The goal of this study is to review the EMR data and consider its health policy implications.

Design: Population-based, retrospective analysis of clinical electronic database.

Datasource: Machshava Briah (“Healthy Thought”) database of MCHC clinical records. This EMR services the MCHCs run by the Ministry of Health, the municipalities of Jerusalem and Tel Aviv and the Leumit Health Fund. This database reflects approximately 70% of Israeli children with a nationwide distribution.

Data Extraction: A list of variables was prepared by researchers in The Maternal and Child Health Department to be extracted from the EMR database for the years 2016 through 2022. The year 2016 indicates the initiation of the EMR use on a nationwide basis. The year 2022 is the latest year with a full year of data available at the time of the study. A team of data analysts from the Digital and Data Technologies Division of the Ministry of Health prepared the data, checking for quality and consistency in their data warehouse.

Data Analysis: Descriptive statistics were prepared including demographic variables (age of mother, population group (sector), residence, parity, length of time between pregnancies), characteristics of the infant (birthweight, gestational age, singleton vs. multiple), and nutrition tracking (exclusive breastfeeding and any breastfeeding). The sector index identifies concentrations of a population, according to the level of presence in each statistical area [8]. The index is updated in accordance with the Central Bureau of Statistics that is published annually. Residence was based on the peripherality index used by the Israeli Central Bureau of Statistics, calculated as the average of standard deviations of two components: (1) the proximity to the local services and weighted by their population size and (2) the proximity to the border of the Tel Aviv district [9]. Comparisons were conducted measuring breastfeeding rates over time and between different sub-groups (SQL V18.9.2, Tableau 2022.3.5).

The representativeness of the study population has been verified by providing rates for the same demographic variables in the general population (see supplementary material– Appendix 1).

The potential for bias in self-reported data and recall bias are addressed in the supplementary material (see Appendix 2 as well as the provision of a structured international comparison on methodologies used in similar studies from Germany, the United Kingdom, Canada and the United States (see Appendix 3).

## Results

The dataset consists of 945,437 records over the study period from 2016 to 2022. Descriptive characteristics of our sample are presented in Table 1.

**Table 1:** Characteristics of study population, infants n = 945,437, mothers n = 932,684, 2016–2022

	2016	2017	2018	2019	2020	2021	2022
Total mothers	134,787	136,507	136,634	133,508	129,448	132,210	129,590
*Maternal characteristics*							
Age (years) %							
< 20	1.8	1.8	1.7	1.5	1.5	1.3	1.3
20–24	19.6	19.5	19.3	19.2	19.1	18.4	18.7
25–29	30.3	30.1	30.1	30.1	30.0	29.9	29.7
30–34	28.3	28.6	28.3	28.1	28.1	29.1	28.7
35–39	15.4	15.4	15.8	16.1	16.1	16.6	16.5
40 +	4.7	4.7	4.8	4.9	4.8	4.8	5.2
*Population group*							
Religious and Secular	59.6	59.1	58.6	58.0	57.7	57.6	56.1
Ultra- Orthodox	24.0	24.0	24.9	25.4	25.6	25.7	26.4
Arab	16.4	16.9	16.5	16.6	16.7	16.7	17.5
*Residence*							
Urban	55.6	54.9	55.0	54.4	54.3	53.7	53.0
Suburban	29.7	30.0	30.1	30.3	30.5	31.0	31.1
Rural	14.8	15.1	14.9	15.3	15.2	15.2	15.9
Primapara	32.4	31.9	31.3	31.2	30.9	29.9	31.4
*Length of time between pregnancies*							
< one year	1.2	1.2	1.3	1.3	1.2	1.2	1.3
1–2 years	21.6	21.7	21.9	22.1	22.0	21.6	20.9
> 2 years	44.8	45.2	45.5	45.4	45.9	47.3	46.4
*Infant characteristics*							
Total infants	136,858	138,578	138,529	135,363	130,969	133,911	131,229
*Birthweight in grams*							
< 1500	0.8	0.7	0.7	0.7	0.6	0.7	0.6
1500–2499	6.8	6.9	6.6	6.6	6.0	6.3	6.5
2500–4000	87.6	87.3	87.6	87.7	88.1	88.0	88.1
> 4000	4.8	5.1	5.1	5.0	5.3	5.0	4.7
*Gestational age (weeks)*							
< 32	0.7	0.7	0.7	0.7	0.7	0.7	0.7
32–35	3.1	3.1	3.1	3.1	2.6	2.9	2.8
36–37	9.7	9.4	10.4	10.5	10.1	10.7	10.9
> 37	86.5	86.8	85.9	85.7	86.6	85.6	85.6
Twins	4.1	4.2	4.0	4.0	3.3	3.5	3.5

**Fig. 1:**
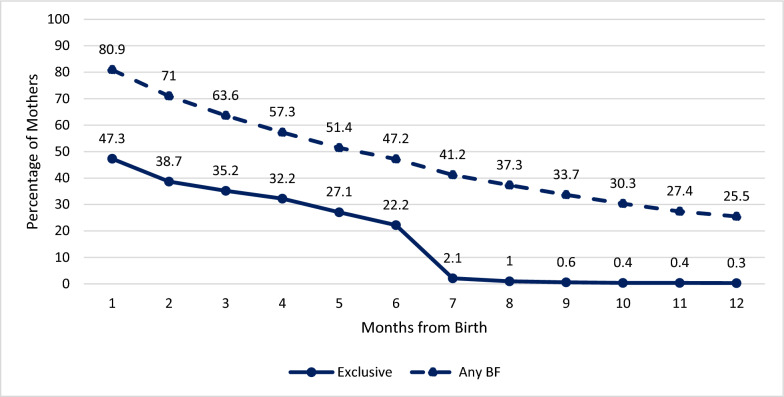
Exclusive breastfeeding and any breastfeeding by age of infant, 2022. n = 131,229

In 2022, the last year for which total data is available, the Any Breastfeeding (ABF) rate at one month, three months, six months, and 12 months was 80.9%, 63.6%, 47.2% and 25.5%, respectively. During this period, over the first half year of life, Exclusive Breastfeeding (EBF) was 47.3% (at one month), 35.2% (at three months), 22.2% (at six months) (Fig. 1). The percentages of women with ABF have shown an overall gradual decline from year to year, as has EBF (see supplemental material, Table 1 A). There was a very slight increase in breastfeeding rates for infants at three months through to 10 months in the year 2020. This phenomenon is documented in an Israeli study of the impact of COVID-19 pandemic on breastfeeding [10]. The differences in breastfeeding rates found by population sector will be addressed in a separate paper.

### Subgroup analyses

#### Birth order

**Fig. 2:**
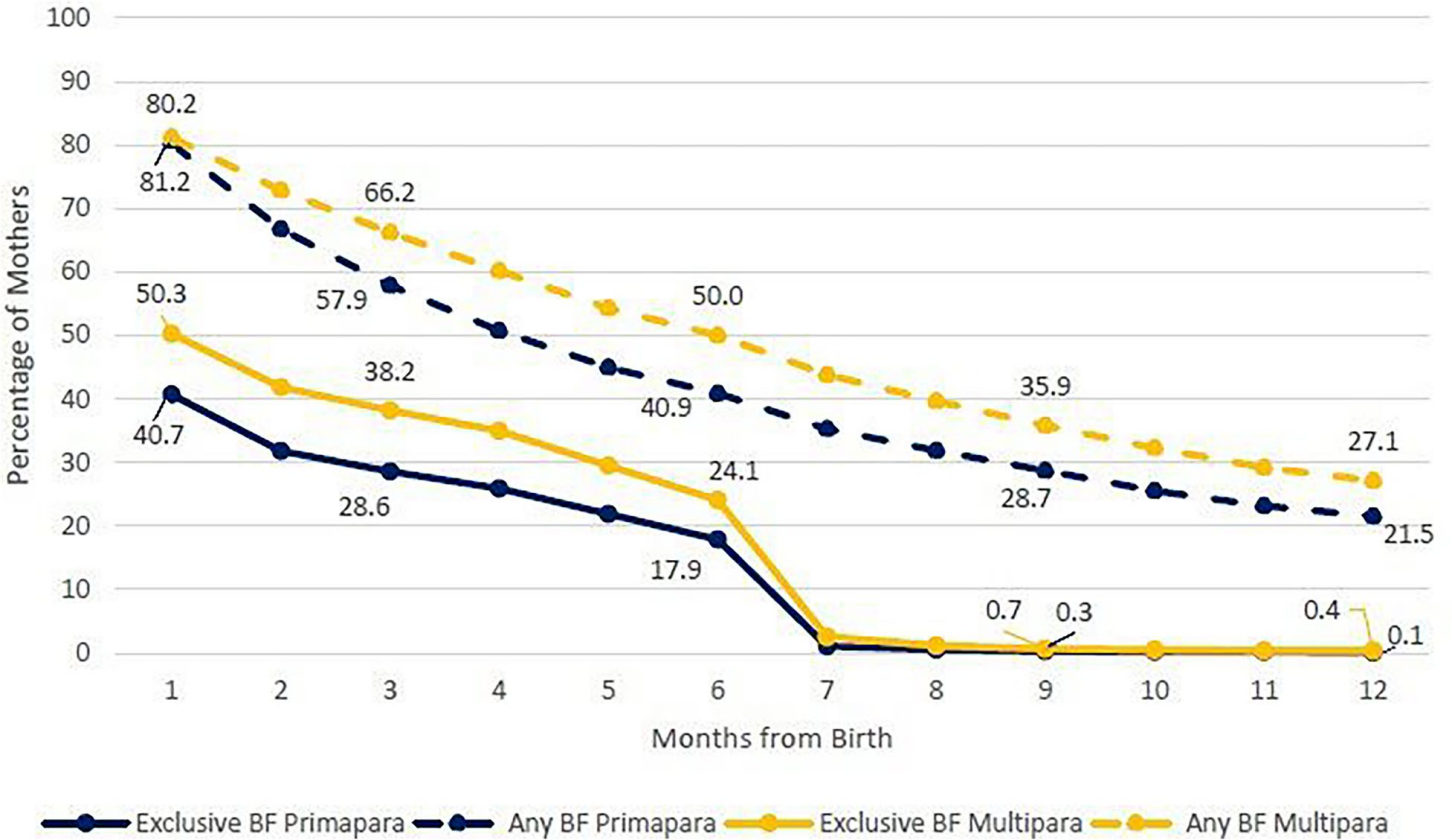
Exclusive breastfeeding and any breastfeeding for primapara and multipara mothers by months from birth, 2022. n = 131,229

In 2022, EBF rates for multipara mothers were higher than for primapara mothers across the study period at one month after birth. For ABF rates at one month after birth, both groups showed similar rates, yet at six months of age, ABF rates were higher in multipara mothers than for primapara mothers (Fig. 2). A gradual decline is detected in this analysis as well (see supplemental material, Table 2 A).

#### Singleton versus twins

**Fig. 3:**
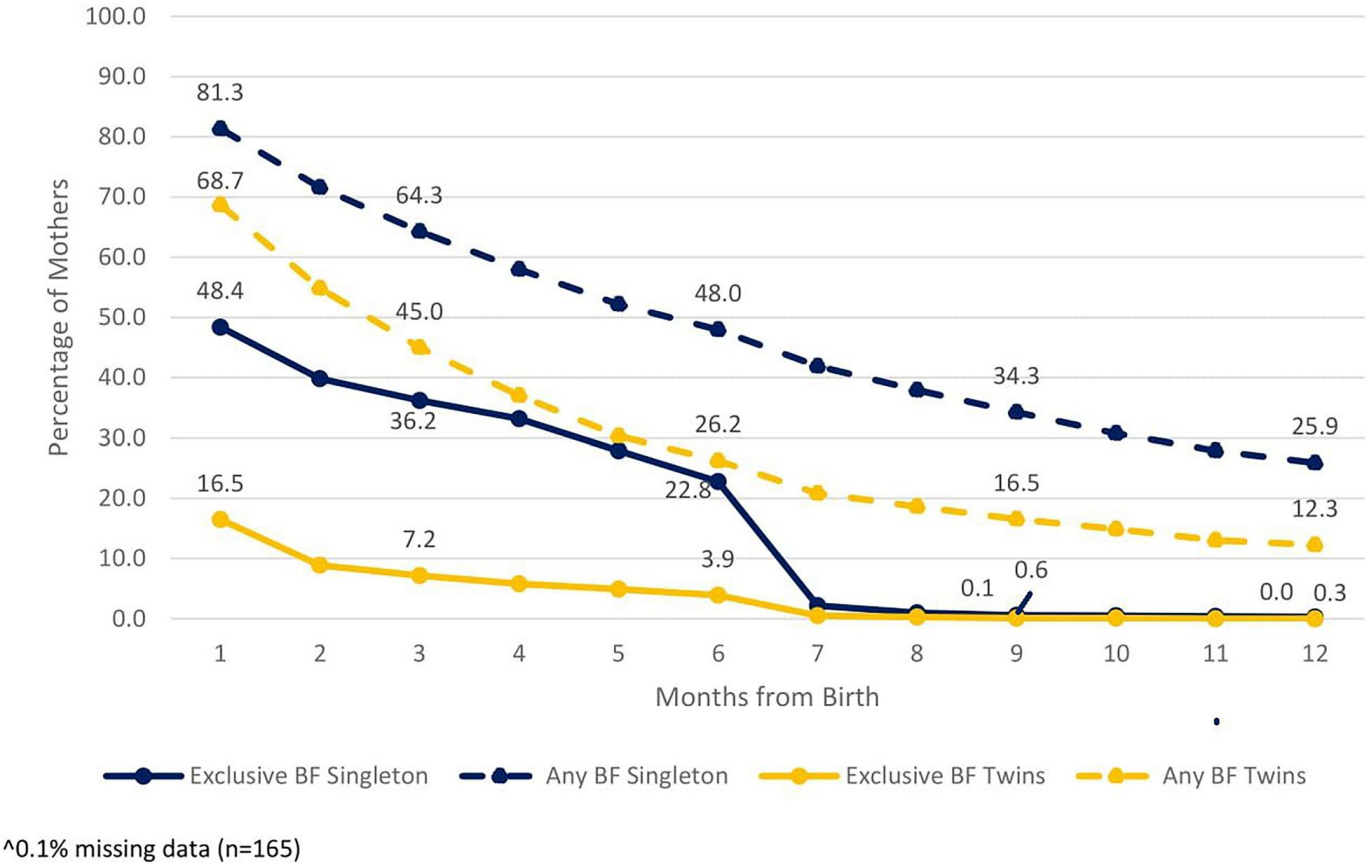
Comparison of Breastfeeding Rates^ for Mothers of Singleton births vs. Mothers of Twin births, 2022. Singleton births n = 126,483, Twin births n = 4,581

Rates of mother’s breastfeeding twins in 2022 are presented in Fig. 3. The EBF rates dropped much faster than the ABF rates. These rates were generally consistent over the entire study period with the exception of the same phenomenon for 2020 as mentioned earlier (see supplemental material, Table 3 A).

#### Premature versus full term

**Fig. 4:**
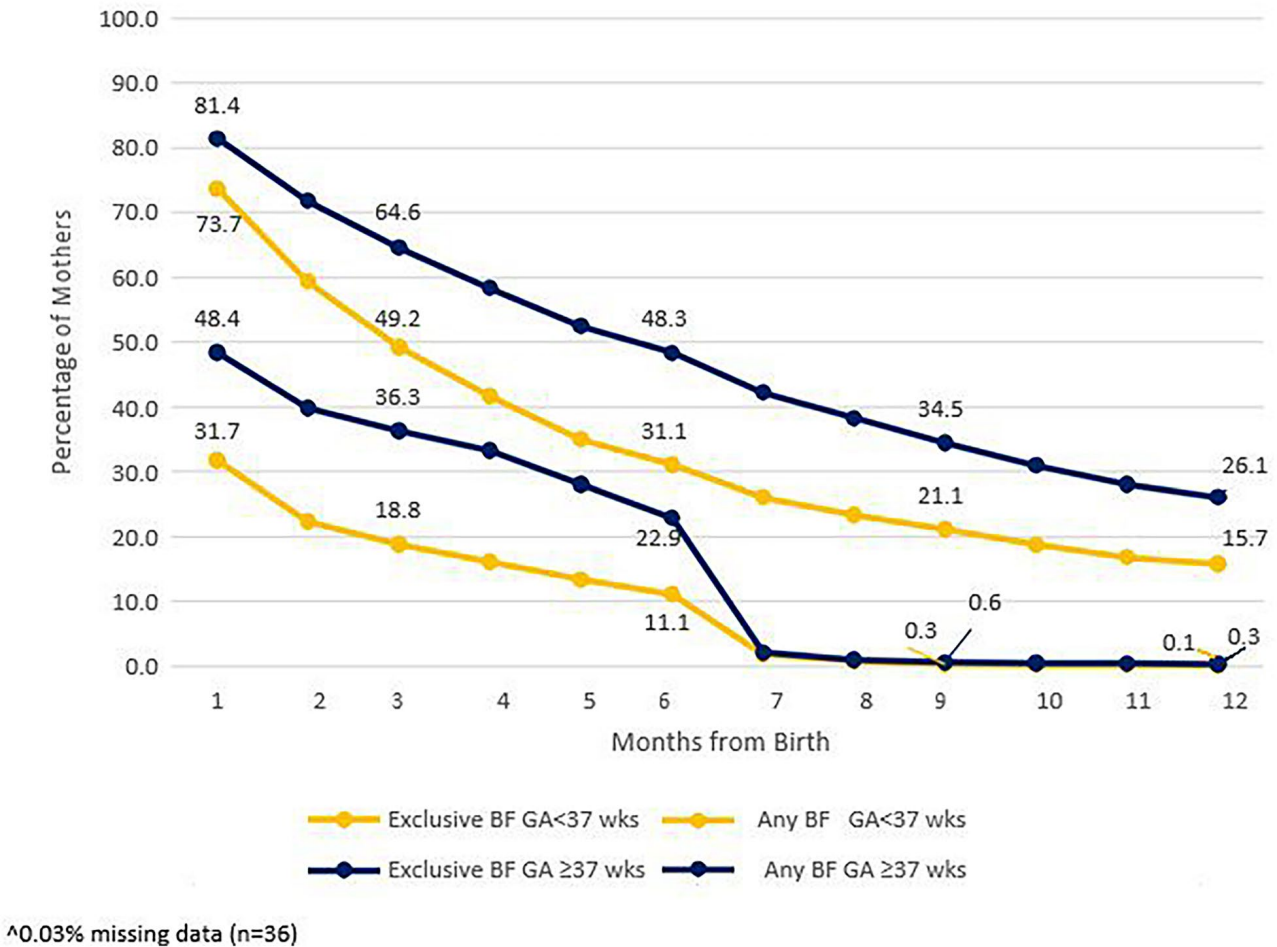
Comparison of breastfeeding rates^ for mothers of premature infants (< 37 weeks) vs. mothers of full term infants (≥ 37 weeks), 2022. Premature infants n =8,524, full term infants n = 122,669

Mothers of preterm infants (< 37 weeks’ gestation) breastfeed less than mothers of full term infants and were less likely to exclusively breastfeed (Fig. 4). The same pattern was seen when the analysis was performed by low birthweight (< 2500 g). This pattern is exhibited over the seven-year period (see supplemental material Fig. 1A and Table 4 A for gestational age).

#### Maternal age

Table 2 shows maternal age was associated with breastfeeding rates. EBF rates among mothers of two-month-old infants were highest for the 20–24 year old age group over the study period (ranging from 45 to 42%) whether or not it was their first birth. The lowest EBF was in the 40 years and above age group with an average of 31%, closely followed by the under 20 year old mothers at average of 32%. ABF appeared similarly with highest rates among 20–24 year old mothers, lowest in 40 plus year old mothers. The other age groups all averaged at 73% for ABF rates across the study period.

**Table 2:** Exclusive breastfeeding and any breastfeeding at two months from birth by maternal age, 2016–2022 n = 945,418.^

Breastfeeding Status	EBF	ABF	EBF	ABF	EBF	ABF	EBF	ABF	EBF	ABF	EBF	ABF
Maternal Age(years)	< 20		20–24		25–29		30–34		35–39		40 +	
2016	35.3	74.1	44.8	78.7	42.4	73.4	43.3	73.6	40.3	73.4	33.9	71.3
2017	34.8	72.9	43.6	77.7	40.4	72.7	41.2	72.4	39.5	73.5	32.2	70.5
2018	34.1	72.6	43.8	77.6	41.8	73.0	41.9	72.9	39.0	73.3	32.0	70.5
2019	34.1	71.2	44.2	77.5	40.7	72.8	41.6	72.3	38.4	72.8	33.3	70.9
2020	32.6	70.8	45.2	77.5	41.6	72.5	41.7	72.2	39.4	72.5	33.3	70.7
2021	32.7	67.8	43.5	76.0	40.3	71.3	39.4	70.4	37.6	71.5	32.0	69.8
2022	25.9	65.0	42.2	74.6	38.9	70.2	39.5	70.5	36.6	70.6	30.6	68.2

^Missing data for 29 cases

When comparing BF rates for mothers of infants at the age of two months for length of time after last pregnancy, we observed the highest rates in mothers whose last pregnancy was 21–23 months from the current birth. Total EBF was 54.9% ranging from 53.7% to 56.0% and total ABF rate of 82%, ranging from 82.9 to 80.7% for the study period. The lowest breastfeeding rates were among mothers whose pregnancy was less than a year from the previous pregnancy (total EBF rate 18.5% ranging from 14.5% to 21.3% and total ABF 50.8%, ranging from 45.2 to 53.0% from 2016 to 2022). The BF rates for exclusive and any breastfeeding increased as the length of time between pregnancies increased up until the 24th month mark where both rates decreased gradually as the length of time increased between pregnancies (See supplemental material, Table 5 A).

#### Residential area

Quite consistently across the study period, mothers residing in urban areas had the highest EBF rates, averaging 53.1% at one month of age compared to mothers residing in suburban and rural areas with 49.8% and 43.8%, respectively. At six months of age, EBF rates were 25.9% for mothers residing in urban areas compared to 20.2% and 15% suburban and rural settings. Yet, ABF rates were higher among mothers residing in rural areas, 83.7% and suburban areas, 81.4%, compared to mothers residing in urban areas with 79.5% at age of one month. At six months of age ABF was 50.4% for mothers living in urban areas, 45.7% in suburban areas. Mothers residing in rural areas had the lowest rates of ABF by six months after birth 40.5% in 2022. (See Table 3). Supplemental material for the full study period is provided in Table 6 A.

**Table 3:** Exclusive breastfeeding and any breastfeeding by residential area (rural, suburban, urban) by months from births, 2022 Rural n = 20,388, Suburban n = 40,058, Urban n = 68,180. ^

Months from Birth	1		2		3		4		5		6		7	8	9	10	11	12
Breastfeeding Status	EBF	ABF	EBF	ABF	EBF	ABF	EBF	ABF	EBF	ABF	EBF	ABF	ABF	ABF	ABF	ABF	ABF	ABF
Residence																		
Rural	39.3	83.7	29.2	71	25.4	61.1	23	52.8	19	45.2	15	40.5	34.7	31.8	28.8	25.8	23.4	21.6
Sub-urban	45.5	81.4	37.2	70.7	33.8	62.6	30.8	55.8	25.5	49.9	20.2	45.7	39.8	36	32.7	29.5	26.9	25.1
Urban	51.4	79.5	43.1	71	39.6	65	36.4	59.7	31.1	54.4	25.9	50.4	44.2	40	36	32.2	29.1	27

^2.5% missing data

## Discussion

This study provides breastfeeding rates in Israel for 2016–2022 from population data based on approximately 70% of national births. Direct comparison with other countries is difficult as data from other countries are based on representative samples. However, with that caveat in mind, some year-specific comparisons can be made. In Germany [11] 2017–2019 and Canada [12] 2017–2018, BF rates were higher for EBF at 68%and 66.9%, yet similar for ABF: at 73%and 90% at two months after birth; in 2019 for the United States, [13] EBF at six months was 24.9% and ABF 55.8%; and 35.9% for ABF at one year. For 2019 in countries surveyed in Western Europe, [14] ABF at six months and one year after birth were 44.5% and 28.5% not unlike to Israel in that year. A UK Study in 2020 of just over 4000 respondents, reported EBF 18.0% and ABF 48.3% at six months [15]. According to a survey of 11 European countries reported by Theurich et al., rates for any breastfeeding at six months ranges from 38 to 71% (exclusive breastfeeding ranged from 14 to 19%), respectively [14].

In our study, multiparas were more likely to breastfeed than primaparas with a similar attrition curve. This is in contradistinction to the findings of Buckman et al. In that study, primiparas were more likely to breastfeed than multiparas, but they had shorter mean breastfeeding duration [16]. In our study, the 20–24 age group had the highest rates both in EBF and Partial BF which on the surface might seem to contradict the finding regarding primaparity. However, this age group was in fact more likely to breastfeed regardless of their primpara or multipara status. This finding may reflect the setting in Israel where many women enter motherhood at young ages. Alternatively, it may indicate renewed interest in breastfeeding among women in this age group.

The breastfeeding rates for Israel are far from the recommendations published for the World Health Organization Global breastfeeding scorecard 2022 with exclusive breastfeeding at age six months for 70% of infants as the target for 2030 [17].

According to UNICEF, the sharp decline in breastfeeding rates has been attributed to several factors, including aggressive marketing campaigns for breastmilk substitutes and the weak implementation of legislation to regulate the practice, a lack of knowledge about the importance of breastfeeding, as well as women returning to work soon after the baby is born [18]. One example of these aggressive tactics is reported for digital marketing whereby formula milk companies are paying social media platforms and influencers to gain direct access to pregnant women and mothers at some of the most vulnerable moments in their lives [19]. In 2023 a full Lancet series featured experts calling for clampdowns on exploitative formula milk marketing [20].

An often-proposed reason for low EBF rates is women’s return to work [21–23]. However, it should be noted that Israel has funded maternity leave of 12–15 weeks in the years studied and much of the drop-off in this study occurs before this time. In a recent study on breastfeeding challenges in Israel, 868 women up to three months post-partum were asked about lactation support in the hospital and post-discharge. The study showed that much of the drop-off in breastfeeding occurred during maternity leave indicating breastfeeding challenges other than employment. Even though initiation rates were impressively high (91.8%), duration was greatly reduced after the first two weeks. Challenges to maintain breastfeeding other than employment were presented as mechanical (i.e., difficulties with latching on and pain), concerns over milk supply and a lack of general support, especially for first time mothers [24]. Breastfeeding support needs of women in hospital and community-based must be addressed.

To address these needs, the National Committee for Promoting Breastfeeding in Israel has prepared a comprehensive strategy report for 2030 [25]. This includes working to implement The International Code of Marketing of Breast-milk Substitutes [26], which limits aggressive marketing of infant foods [27]. Enforcing the code is included in the 10 Steps for promoting and protecting breastfeeding as stated in the Baby Friendly Hospital Initiative (BFHI), an evidence-based, international policy shown to positively impact on breastfeeding duration [28]. DiGirolamo’s study of mothers who intended to breastfeed for more than two months stopped breastfeeding before six weeks when the number of “Baby-Friendly” practices reported in the hospital were lower than six of the Ten Steps [23].

Israel’s current hospital breastfeeding policy is based on the 1992 version of the Baby Friendly Hospital Initiative [29]. However, this policy is not consistently enforced. Steps are being put into place to improve adherence including a quality indicator of exclusive breastfeeding at hospital discharge. Strides are being taken in the community as well. Published in October of 2022 and updated in July, 2023 are the guidelines for staff working in the MCHCs [30]. The purpose of the guidelines is to regulate the support and guidance provided on the subject of breastfeeding within the framework of the MCHC in accordance with up-to-date professional and scientific information. The staff provides relevant information while referring to the needs of the child, the wishes of the mother and the family. There is a telephone hotline during weekday afternoons through to the evening hours and on Fridays for mothers to call and consult with a nurse, nutritionist or a specialized counselor on well-child care including breastfeeding concerns (established in February, 2020).

This study is the first of its kind for Israel, providing up-to-date, national, population-based breastfeeding rates for close to a million infant records over seven years. This will provide the baseline against which future interventions will be measured.

### Limitations

The information on breastfeeding rates in the EMR database is based on maternal reports which is the standard, but not immune from recall bias. This study does not describe differences in BF rates by ethnicity (Arab/Jewish) as there is universal health care for all, and due to the considerable diversity, socioeconomic and cultural, within each major ethnic group, this analysis will be offered in a future study.

The data presented here does not include breastfeeding rates from hospital data. It is important to collect information on initiation of breastfeeding and numbers of women breastfeeding upon release from the hospital, and whether supplements were introduced during their hospital stay. It is anticipated that the data from the quality indicator mentioned above will provide this information in the fore-seeable future.

## Conclusions

The population-based data gives us an important measure for a baseline marker. This study shows drops in BF rates indicating a need to investigate reasons for discontinuing BF and identifying possible areas for offering support. Further research is required in order to establish effective health policy in the hospitals as well as in the community for women who lack the necessary support. The National BF Promotion Committee has documented the relevant steps needed to expand on current health policy and engage various members in the community to join in the mission of creating a mother and baby-friendly, family-friendly environment in Israel.

Our policy recommendations fall into three categories:

1. Improving information available to the public, potential parents, their supporters (family/friends) and all staff who have contact with this audience
2. Expanding sources of support for those who choose to breastfeed
3. Establish new policy to protect, promote and support breastfeeding

The first two goals have been implemented as seen on Ministry of Health web site [31] – serving as a rich resource (offered in four languages) in the parenting section on breastfeeding including the significance of breastfeeding, guide to breastfeeding and strategies for overcoming breastfeeding challenges. Another essential step is to educate the public on the known risks of using infant formula in place of breast milk: Increased gastrointestinal diseases, including necrotizing enterocolitis [32–34]. Increased infectious diseases, including respiratory tract infection. Altered adiposity and intellectual development. Increased maternal breast cancer through reduced duration of breastfeeding [35] Adverse effects related to formula contamination or reconstitution problems—eg, bacterial infection or burn injury. Increased cost of purchasing milk.

Also, as mentioned earlier, the Ministry of Health (Kol Habriut) hotline offers standardized breastfeeding advice by lactation consultants through MCHC afternoons, evenings, and on Fridays, free of charge.

Around the world, the process that has proven most successful in protecting, promoting, and supporting breastfeeding is the Baby Friendly Hospital Initiative [21, 36, 37]. A work force from the Ministry of Health and professional organizations is currently building the accreditation for this initiative in Israel according to the principles of global BFHI. This process is based on a review of literature, participation in international conferences, and through peer learning as members of the global Baby Friendly Hospital Network [38]. The process includes site visits to all hospitals in Israel offering maternity services.

In our attempts to overcome potential barriers, proposals are being reviewed. One suggestion was found in a recent article in the International Breastfeeding Journal [39] presented a systematic review of five academic databases (57 articles) whereby the research question was ‘What are the outcomes of implementing the national law to regulate commercial milk formula (CMF) promotions in countries where the Code was legislated into law?’ The conclusion stated the need to enhance legal compliance and establish dedicated monitoring and reporting systems. Since human resources are limited in Israel, technology-assisted solutions for monitoring compliance can be an option. Education for health workers on communication strategies regarding national policy are also essential in enhancing their acceptability and compliance.

## Supplementary Information

Supplementary material 1

Supplementary material 2

Supplementary material 3

Supplementary material 4

Supplementary material 5

Supplementary material 6

Supplementary material 7

Supplementary material 8

Supplementary material 9

## Data Availability

The data that support the findings of this study are not openly available due to reasons of sensitivity and are available from the corresponding author upon reasonable request.

## Abbreviations

BF: Breastfeeding
BFHI: Baby friendly hospital initiative
EBF: Exclusive breastfeeding
ABF: Any breastfeeding
MCHC: Maternal and child health clinic
EMR: Electronic medical record
