# Supplementary material for: Breastfeeding rates in Israel and their health policy implications
Source: Isr J Health Policy Res. 2025 May 13;14:28. doi: 10.1186/s13584-025-00689-1 (PMC12077002; doi:10.1186/s13584-025-00689-1)
Supplement: Supplementary file 1 — Supplementary material 1 [file 13584_2025_689_MOESM1_ESM.docx]

Supplementary Table 1A, Additional File 1

**Exclusive Breastfeeding and Any Breastfeeding among Mothers for 2016-2022, by Month from Birth. n=945,437 infant records.**

| Month      Year | **1** | | **2** | | **3** | | **4** | | **5** | | **6** | | **7*** | **8** | **9** | **10** | **11** | **12** |
| --- | --- | --- | --- | --- | --- | --- | --- | --- | --- | --- | --- | --- | --- | --- | --- | --- | --- | --- |
|  | **EBF** | **ABF** | **EBF** | **ABF** | **EBF** | **ABF** | **EBF** | **ABF** | **EBF** | **ABF** | **EBF** | **ABF** | **ABF** | **ABF** | **ABF** | **ABF** | **ABF** | **ABF** |
| **2016** | 54.1 | 83.8 | 42.3 | 74.4 | 37.6 | 67.2 | 33.7 | 60.7 | 25.5 | 54.6 | 20.0 | 50.2 | 43.7 | 40.0 | 36.1 | 32.5 | 29.5 | 27.2 |
| **2017** | 51.6 | 83.4 | 40.6 | 73.6 | 36.3 | 66.1 | 32.9 | 59.7 | 25.0 | 53.7 | 19.4 | 49.5 | 43.4 | 39.7 | 35.9 | 32.4 | 29.5 | 27.3 |
| **2018** | 51.2 | 83.3 | 41.2 | 73.8 | 36.9 | 66.4 | 33.7 | 60.2 | 26.2 | 54.1 | 20.5 | 49.9 | 43.6 | 39.9 | 36.2 | 32.7 | 29.7 | 27.4 |
| **2019** | 50.3 | 82.9 | 40.8 | 73.4 | 36.6 | 65.9 | 33.7 | 59.8 | 27.0 | 54.0 | 21.4 | 49.9 | 44.0 | 40.6 | 37.1 | 33.9 | 31.1 | 29.1 |
| **2020** | 50.2 | 82.6 | 41.5 | 73.3 | 37.7 | 66.0 | 34.9 | 60.0 | 28.2 | 54.3 | 22.3 | 50.3 | 44.2 | 40.8 | 37.3 | 34.0 | 31.1 | 29.0 |
| **2021** | 48.4 | 81.3 | 39.7 | 71.8 | 35.8 | 64.1 | 33.0 | 58.0 | 27.5 | 52.2 | 22.5 | 48.1 | 42.1 | 38.6 | 35.1 | 31.8 | 29.0 | 27.0 |
| **2022** | 47.3 | 80.9 | 38.7 | 71.0 | 35.2 | 63.6 | 32.2 | 57.3 | 27.1 | 51.4 | 22.2 | 47.2 | 41.2 | 37.3 | 33.7 | 30.3 | 27.4 | 25.5 |

*EBF is recommended for the first six months; therefore, we present only ABF from 7-12 months.
