# Supplementary material for: Breastfeeding rates in Israel and their health policy implications
Source: Isr J Health Policy Res. 2025 May 13;14:28. doi: 10.1186/s13584-025-00689-1 (PMC12077002; doi:10.1186/s13584-025-00689-1)
Supplement: Supplementary file 2 — Supplementary material 2 [file 13584_2025_689_MOESM2_ESM.docx]

Supplementary Table 2A, Additional File 2

**Any Breastfeeding for Primapara and Multipara Mothers by Months from Birth, 2016-2022. N=944,196.^**

| **Month**  **Year** | **1** | | **2** | | **3** | | **4** | | **5** | | **6** | | **7*** | **8** | **9** | **10** | **11** | **12** |
| --- | --- | --- | --- | --- | --- | --- | --- | --- | --- | --- | --- | --- | --- | --- | --- | --- | --- | --- |
| Primapara | EBF | ABF | EBF | ABF | EBF | ABF | EBF | ABF | EBF | ABF | EBF | ABF | ABF | ABF | ABF | ABF | ABF | ABF |
| **2016** | 48.5 | 83.2 | 36.2 | 70.6 | 31.9 | 61.7 | 28.5 | 54.2 | 21.1 | 47.6 | 16.3 | 43.2 | 36.8 | 33.0 | 29.5 | 26.1 | 23.5 | 21.5 |
| **2017** | 45.8 | 82.6 | 34.4 | 69.4 | 30.4 | 60.4 | 27.2 | 53.0 | 20.3 | 46.7 | 15.5 | 42.4 | 36.5 | 32.8 | 29.1 | 25.9 | 23.4 | 21.3 |
| **2018** | 45.5 | 82.9 | 34.6 | 69.8 | 30.6 | 60.6 | 27.7 | 53.5 | 21.3 | 47.2 | 16.4 | 43.0 | 36.9 | 33.1 | 29.7 | 26.3 | 23.7 | 21.7 |
| **2019** | 44.4 | 82.3 | 34.4 | 69.5 | 30.5 | 60.6 | 27.8 | 53.7 | 22.0 | 47.6 | 17.3 | 43.3 | 37.4 | 33.9 | 30.7 | 27.6 | 25.1 | 23.3 |
| **2020** | 44.4 | 82.5 | 35.0 | 69.8 | 31.5 | 60.9 | 28.8 | 54.0 | 23.2 | 48.3 | 18.1 | 44.3 | 38.1 | 34.9 | 31.4 | 28.3 | 25.7 | 23.9 |
| **2021** | 42.6 | 81.1 | 33.9 | 68.4 | 30.3 | 59.2 | 27.7 | 52.3 | 23.1 | 46.4 | 18.9 | 42.4 | 36.6 | 33.2 | 29.8 | 26.6 | 24.1 | 22.3 |
| **2022** | 40.7 | 80.2 | 31.8 | 66.8 | 28.6 | 57.9 | 25.9 | 50.8 | 21.9 | 45.0 | 17.9 | 40.9 | 35.3 | 31.9 | 28.7 | 25.6 | 23.2 | 21.5 |
|  |  |  |  |  |  |  |  |  |  |  |  |  |  |  |  |  |  |  |
| Multipara | EBF | ABF | EBF | ABF | EBF | ABF | EBF | ABF | EBF | ABF | EBF | ABF | ABF | ABF | ABF | ABF | ABF | ABF |
| **2016** | 56.8 | 84.1 | 45.2 | 76.2 | 40.4 | 69.8 | 36.2 | 63.8 | 27.6 | 57.9 | 21.7 | 53.5 | 47.0 | 43.3 | 39.3 | 35.6 | 32.3 | 30.0 |
| **2017** | 54.3 | 83.7 | 43.5 | 75.6 | 39.1 | 68.8 | 35.6 | 62.9 | 27.2 | 57.0 | 21.3 | 52.8 | 46.6 | 43.0 | 39.0 | 35.5 | 32.3 | 30.0 |
| **2018** | 53.8 | 83.4 | 44.1 | 75.6 | 39.7 | 69.0 | 36.4 | 63.2 | 28.4 | 57.2 | 22.3 | 53.0 | 46.6 | 43.0 | 39.2 | 35.6 | 32.4 | 30.0 |
| **2019** | 53.0 | 83.2 | 43.7 | 75.2 | 39.4 | 68.3 | 36.4 | 62.6 | 29.2 | 56.9 | 23.3 | 52.9 | 46.9 | 43.6 | 40.1 | 36.8 | 33.8 | 31.7 |
| **2020** | 52.8 | 82.7 | 44.3 | 74.8 | 40.5 | 68.3 | 37.7 | 62.7 | 30.4 | 57.0 | 24.1 | 53.1 | 46.9 | 43.5 | 39.9 | 36.5 | 33.5 | 31.3 |
| **2021** | 50.8 | 81.5 | 42.2 | 73.3 | 38.2 | 66.2 | 35.2 | 60.4 | 29.3 | 54.7 | 24.0 | 50.6 | 44.4 | 40.9 | 37.4 | 34.0 | 31.0 | 29.0 |
| **2022** | 50.3 | 81.2 | 41.9 | 72.9 | 38.2 | 66.2 | 35.0 | 60.2 | 29.5 | 54.3 | 24.1 | 50.0 | 43.8 | 39.7 | 35.9 | 32.3 | 29.2 | 27.1 |

^0.13% missing data (n=1,241)
