# Supplementary material for: Breastfeeding rates in Israel and their health policy implications
Source: Isr J Health Policy Res. 2025 May 13;14:28. doi: 10.1186/s13584-025-00689-1 (PMC12077002; doi:10.1186/s13584-025-00689-1)
Supplement: Supplementary file 3 — Supplementary material 3 [file 13584_2025_689_MOESM3_ESM.docx]

Supplementary Table 3A, Additional File 3

**Exclusive Breastfeeding and Any Breastfeeding for Singleton births and Twin births by months after birth, 2016-2022. N=944,196.^**

| **Month**  **Year** | **1** | | **2** | | **3** | | **4** | | **5** | | **6** | | **7** | **8** | **9** | **10** | **11** | **12** |
| --- | --- | --- | --- | --- | --- | --- | --- | --- | --- | --- | --- | --- | --- | --- | --- | --- | --- | --- |
| **Singles** | EBF | ABF | EBF | ABF | EBF | ABF | EBF | ABF | EBF | ABF | EBF | ABF | ABF | ABF | ABF | ABF | ABF | ABF |
| **2016** | 55.8 | 84.4 | 43.8 | 75.2 | 39.0 | 68.1 | 35.0 | 61.7 | 26.4 | 55.6 | 20.7 | 51.2 | 44.6 | 40.9 | 36.9 | 33.3 | 30.2 | 27.9 |
| **2017** | 53.1 | 83.9 | 42.1 | 74.4 | 37.6 | 67.0 | 34.2 | 60.7 | 25.9 | 54.7 | 20.1 | 50.5 | 44.4 | 40.6 | 36.8 | 33.2 | 30.2 | 27.9 |
| **2018** | 52.6 | 83.8 | 42.5 | 74.5 | 38.1 | 67.2 | 34.8 | 61.1 | 27.0 | 55.0 | 21.1 | 50.9 | 44.5 | 40.8 | 37.0 | 33.4 | 30.4 | 28.0 |
| **2019** | 51.7 | 83.4 | 42.1 | 74.2 | 37.8 | 66.8 | 34.9 | 60.8 | 27.9 | 55.0 | 22.1 | 50.9 | 44.9 | 41.4 | 38.0 | 34.7 | 31.8 | 29.7 |
| **2020** | 51.4 | 83.0 | 42.6 | 73.8 | 38.7 | 66.7 | 35.9 | 60.7 | 29.0 | 55.1 | 22.9 | 51.1 | 44.9 | 41.5 | 37.9 | 34.6 | 31.6 | 29.6 |
| **2021** | 49.6 | 81.8 | 40.8 | 72.5 | 36.9 | 64.9 | 34.0 | 58.8 | 28.3 | 53.0 | 23.1 | 49.0 | 42.9 | 39.4 | 35.9 | 32.5 | 29.6 | 27.6 |
| **2022** | 48.4 | 81.3 | 39.8 | 71.6 | 36.2 | 64.3 | 33.2 | 58.0 | 27.9 | 52.2 | 22.8 | 48.0 | 41.9 | 37.9 | 34.3 | 30.8 | 27.9 | 25.9 |
|  |  |  |  |  |  |  |  |  |  |  |  |  |  |  |  |  |  |  |
| **Twins** | EBF | ABF | EBF | ABF | EBF | ABF | EBF | ABF | EBF | ABF | EBF | ABF | ABF | ABF | ABF | ABF | ABF | ABF |
| **2016** | 17.7 | 70.6 | 9.5 | 57.2 | 7.3 | 46.8 | 6.3 | 38.8 | 5.2 | 32.0 | 4.1 | 28.1 | 22.5 | 20.1 | 17.5 | 16.0 | 14.5 | 13.2 |
| **2017** | 16.8 | 71.9 | 8.9 | 57.5 | 7.2 | 47.2 | 6.5 | 39.0 | 5.2 | 32.7 | 4.0 | 28.0 | 22.6 | 19.6 | 17.0 | 14.9 | 13.4 | 12.4 |
| **2018** | 19.7 | 72.2 | 12.0 | 57.9 | 10.0 | 47.7 | 8.6 | 39.9 | 6.8 | 33.0 | 5.3 | 28.7 | 23.5 | 20.7 | 18.4 | 16.1 | 14.5 | 13.3 |
| **2019** | 16.9 | 70.7 | 9.6 | 54.8 | 7.5 | 44.3 | 6.6 | 37.5 | 5.5 | 30.3 | 4.4 | 26.4 | 22.0 | 19.6 | 17.6 | 15.6 | 14.3 | 13.2 |
| **2020** | 18.9 | 72.9 | 11.5 | 59.1 | 9.3 | 48.5 | 8.0 | 41.3 | 6.5 | 34.8 | 5.3 | 31.0 | 25.3 | 22.7 | 20.1 | 17.9 | 16.3 | 14.9 |
| **2021** | 17.0 | 69.7 | 9.4 | 55.0 | 7.3 | 44.2 | 6.7 | 36.0 | 5.5 | 29.8 | 4.6 | 25.8 | 19.5 | 17.5 | 15.5 | 13.8 | 12.4 | 11.4 |
| **2022** | 16.5 | 68.7 | 8.9 | 54.8 | 7.2 | 45.0 | 5.8 | 37.0 | 4.9 | 30.3 | 3.9 | 26.2 | 20.8 | 18.6 | 16.5 | 14.9 | 13.1 | 12.3 |

^Missing data for 18 cases
