# Supplementary material for: Breastfeeding rates in Israel and their health policy implications
Source: Isr J Health Policy Res. 2025 May 13;14:28. doi: 10.1186/s13584-025-00689-1 (PMC12077002; doi:10.1186/s13584-025-00689-1)
Supplement: Supplementary file 4 — Supplementary material 4 [file 13584_2025_689_MOESM4_ESM.docx]

Supplementary Table 4A, Additional File 5

**Exclusive Breastfeeding and Any Breastfeeding by Gestational Age <37 vs ≥37 weeks by Year of Birth, 2016-2022. N=929,900***^*

| **Month**  **Year** | **1** | | **2** | | **3** | | **4** | | **5** | | **6** | | **7** | **8** | **9** | **10** | **11** | **12** |
| --- | --- | --- | --- | --- | --- | --- | --- | --- | --- | --- | --- | --- | --- | --- | --- | --- | --- | --- |
| **<37w** | EBF | ABF | EBF | ABF | EBF | ABF | EBF | ABF | EBF | ABF | EBF | ABF | ABF | ABF | ABF | ABF | ABF | ABF |
| **2016** | 35.6 | 76.5 | 24.7 | 63.3 | 20.3 | 53.1 | 17.6 | 45.0 | 13.5 | 38.7 | 10.7 | 34.0 | 28.2 | 24.9 | 22.2 | 20.0 | 18.0 | 16.6 |
| **2017** | 33.3 | 76.3 | 22.4 | 62.3 | 18.5 | 52.1 | 16.4 | 44.0 | 12.7 | 37.7 | 9.9 | 33.0 | 27.6 | 24.6 | 21.7 | 19.2 | 17.5 | 16.3 |
| **2018** | 33.8 | 76.1 | 23.4 | 61.7 | 19.6 | 51.6 | 17.2 | 44.0 | 13.5 | 37.2 | 10.6 | 33.3 | 27.7 | 24.7 | 21.9 | 19.8 | 17.7 | 16.4 |
| **2019** | 33.6 | 75.6 | 23.8 | 61.1 | 20.0 | 51.3 | 17.4 | 43.6 | 14.2 | 36.9 | 11.5 | 33.0 | 27.8 | 25.3 | 22.8 | 20.5 | 18.6 | 17.2 |
| **2020** | 32.6 | 76.5 | 23.6 | 62.9 | 20.0 | 52.5 | 17.5 | 45.0 | 14.3 | 38.6 | 11.4 | 34.4 | 28.6 | 26.0 | 23.3 | 21.1 | 19.1 | 17.8 |
| **2021** | 32.0 | 74.3 | 25.5 | 60.7 | 18.7 | 50.4 | 16.5 | 42.7 | 13.6 | 36.3 | 11.4 | 32.2 | 26.7 | 24.0 | 21.5 | 19.2 | 17.3 | 16.1 |
| **2022** | 31.7 | 73.7 | 26.1 | 59.3 | 18.8 | 49.2 | 16.1 | 41.6 | 13.4 | 35.0 | 11.1 | 31.1 | 26.1 | 23.3 | 21.1 | 18.7 | 16.8 | 15.7 |
|  |  |  |  |  |  |  |  |  |  |  |  |  |  |  |  |  |  |  |
| **≥37w** | EBF | ABF | EBF | ABF | EBF | ABF | EBF | ABF | EBF | ABF | EBF | ABF | ABF | ABF | ABF | ABF | ABF | ABF |
| **2016** | 55.4 | 84.5 | 43.5 | 75.3 | 38.8 | 68.3 | 34.8 | 61.9 | 26.2 | 55.8 | 20.5 | 51.4 | 44.9 | 41.1 | 37.1 | 33.5 | 30.3 | 28.0 |
| **2017** | 52.8 | 84.1 | 41.8 | 74.6 | 37.4 | 67.2 | 34.0 | 60.9 | 25.7 | 54.9 | 19.9 | 50.7 | 44.5 | 40.8 | 36.9 | 33.3 | 30.3 | 28.0 |
| **2018** | 52.5 | 83.8 | 42.4 | 74.6 | 38.1 | 67.4 | 34.8 | 61.3 | 27.1 | 55.3 | 21.2 | 51.1 | 44.7 | 41.0 | 37.2 | 33.6 | 30.5 | 28.2 |
| **2019** | 51.5 | 83.4 | 42.0 | 74.3 | 37.8 | 67.0 | 34.9 | 61.0 | 27.9 | 55.2 | 22.1 | 51.1 | 45.1 | 41.7 | 38.2 | 34.9 | 32.0 | 29.9 |
| **2020** | 51.4 | 83.0 | 42.6 | 74.0 | 38.9 | 66.9 | 36.1 | 61.0 | 29.1 | 55.4 | 23.0 | 51.4 | 45.2 | 41.8 | 38.2 | 34.8 | 31.9 | 29.8 |
| **2021** | 49.6 | 81.9 | 40.9 | 72.6 | 37.1 | 65.1 | 34.1 | 59.1 | 28.5 | 53.3 | 23.3 | 49.3 | 43.2 | 39.6 | 36.1 | 32.7 | 29.8 | 27.8 |
| **2022** | 48.4 | 81.4 | 39.8 | 71.8 | 36.3 | 64.6 | 33.3 | 58.3 | 28.0 | 52.5 | 22.9 | 48.3 | 42.2 | 38.2 | 34.5 | 31.0 | 28.1 |  |

^.46% Missing data
