# Supplementary material for: Breastfeeding rates in Israel and their health policy implications
Source: Isr J Health Policy Res. 2025 May 13;14:28. doi: 10.1186/s13584-025-00689-1 (PMC12077002; doi:10.1186/s13584-025-00689-1)
Supplement: Supplementary file 5 — Supplementary material 5 [file 13584_2025_689_MOESM5_ESM.docx]

Supplementary Table 5A, Additional File 6

**Exclusive and Partial Breastfeeding Rates by Length of Time between Pregnancies at Two Months after Current Birth, 2022. n=131,229**

| Length of time between pregnancies (months) | 2022 | |
| --- | --- | --- |
|  | EBF | ABF |
| First pregnancy | 31.8 | 66.8 |
| Less than one year | 14.5 | 45.2 |
| 12-14 | 31.2 | 63.5 |
| 15-17 | 42.3 | 72.2 |
| 18-20 | 49.9 | 78.1 |
| 21-23 | 55.5 | 81.9 |
| 24-29 | 51.4 | 78.6 |
| 30-35 | 47.2 | 76.3 |
| 36-41 | 41.5 | 73.1 |
| 42-47 | 38.5 | 71.2 |
| 48-53 | 35.4 | 68.6 |
| 54-59 | 33.2 | 67.7 |
| 5 or more years | 27.5 | 65.7 |
