# Supplementary figures and images for: Breastfeeding rates in Israel and their health policy implications
Source: Isr J Health Policy Res. 2025 May 13;14:28. doi: 10.1186/s13584-025-00689-1 (PMC12077002; doi:10.1186/s13584-025-00689-1)

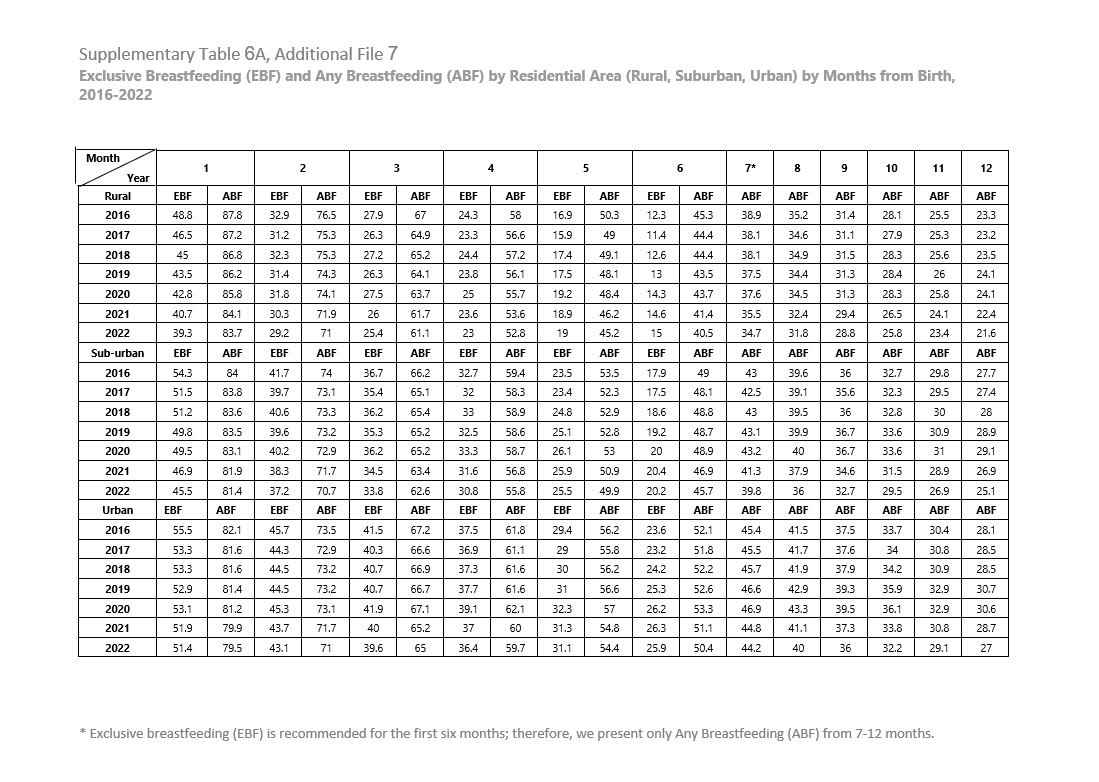

Supplement: Supplementary file 6 — Supplementary material 6 [file 13584_2025_689_MOESM6_ESM.docx]
