# Supplementary material for: Breastfeeding rates in Israel and their health policy implications
Source: Isr J Health Policy Res. 2025 May 13;14:28. doi: 10.1186/s13584-025-00689-1 (PMC12077002; doi:10.1186/s13584-025-00689-1)
Supplement: Supplementary file 7 — Supplementary material 7 [file 13584_2025_689_MOESM7_ESM.docx]

Supplementary material

Appendix 1

**Table 1. Characteristics of Study Population, infants n=945,437, mothers n= 932,684, 2016-2022. General population figures in References column.**

|  | **2016** | **2017** | **2018** | **2019** | **2020** | **2021** | **2022** | **References** |
| --- | --- | --- | --- | --- | --- | --- | --- | --- |
| **Total mothers** | 134,787 | 136,507 | 136,634 | 133,508 | 129,448 | 132,210 | 129,590 |  |
| **Maternal characteristics** | | | | | | | |  |
| **Age (years) %** | | | | | | | | **BI 2022^a^** |
| **<20** | 1.8 | 1.8 | 1.7 | 1.5 | 1.5 | 1.3 | 1.3 | 0.8 |
| **20-24** | 19.6 | 19.5 | 19.3 | 19.2 | 19.1 | 18.4 | 18.7 | 15.6 |
| **25-29** | 30.3 | 30.1 | 30.1 | 30.1 | 30.0 | 29.9 | 29.7 | 27.3 |
| **30-34** | 28.3 | 28.6 | 28.3 | 28.1 | 28.1 | 29.1 | 28.7 | 30.2 |
| **35-39** | 15.4 | 15.4 | 15.8 | 16.1 | 16.1 | 16.6 | 16.5 | 19.4 |
| **40+** | 4.7 | 4.7 | 4.8 | 4.9 | 4.8 | 4.8 | 5.2 | 6.8 |
| **Population group** | | | | | | | | **IDI 2021-2022^b^** |
| **Religious**  **& Secular** | 59.6 | 59.1 | 58.6 | 58.0 | 57.7 | 57.6 | 56.1 | 66% |
| **Ultra-**  **Orthodox** | 24.0 | 24.0 | 24.9 | 25.4 | 25.6 | 25.7 | 26.4 | 13% |
| **Arab** | 16.4 | 16.9 | 16.5 | 16.6 | 16.7 | 16.7 | 17.5 | 21% |
| **Residence** | | | | | | | | **2017^c^** |
| **Urban** | 55.6 | 54.9 | 55.0 | 54.4 | 54.3 | 53.7 | 53.0 | 59.5 |
| **Suburban** | 29.7 | 30.0 | 30.1 | 30.3 | 30.5 | 31.0 | 31.1 | 20.7 |
| **Rural** | 14.8 | 15.1 | 14.9 | 15.3 | 15.2 | 15.2 | 15.9 | 19.7 |
| **Primapara** | 32.4 | 31.9 | 31.3 | 31.2 | 30.9 | 29.9 | 31.4 | 28.6^d^ |
| **Length of time between pregnancies** | | | | | | | | **2016^e^** |
| **< one year** | 1.2 | 1.2 | 1.3 | 1.3 | 1.2 | 1.2 | 1.3 | 25.9 |
| **1-2 years** | 21.6 | 21.7 | 21.9 | 22.1 | 22.0 | 21.6 | 20.9 | 28.2 |
| **>2 years** | 44.8 | 45.2 | 45.5 | 45.4 | 45.9 | 47.3 | 46.4 | 46 |
| **Infant characteristics** | | | | | | | |  |
| **Total infants** | 136,858 | 138,578 | 138,529 | 135,363 | 130,969 | 133,911 | 131,229 |  |
| **Birthweight in grams** | | | | | | | | **2022^f^** |
| **<1500** | 0.8 | 0.7 | 0.7 | 0.7 | 0.6 | 0.7 | 0.6 | 0.7 |
| **1500-2499** | 6.8 | 6.9 | 6.6 | 6.6 | 6.0 | 6.3 | 6.5 | 6.4 |
| **2500-4000** | 87.6 | 87.3 | 87.6 | 87.7 | 88.1 | 88.0 | 88.1 | 88 |
| **>4000** | 4.8 | 5.1 | 5.1 | 5.0 | 5.3 | 5.0 | 4.7 | 4.8 |
| **Gestational age (weeks)** | | | | | | | | **2022^f^** |
| **<28** | 0.2 | 0.1 | 0.2 | 0.2 | 0.1 | 0.2 | 0.1 | 0.2 |
| **28-32** | 0.9 | 0.9 | 0.8 | 0.9 | 0.8 | 0.8 | 0.8 | 0.8 |
| **33-36** | 5.6 | 5.8 | 5.7 | 5.7 | 5.2 | 5.6 | 5.5 | 5.5 |
| **37-41** | 89.0 | 88.8 | 90.5 | 90.4 | 91.2 | 91.0 | 91.0 | 91.0 |
| **42+** | 2.9 | 2.8 | 2.8 | 2.9 | 2.7 | 2.3 | 2.5 | 2.5 |
| **Twins** | 4.1 | 4.2 | 4.0 | 4.0 | 3.3 | 3.5 | 3.5 | 3.6^d^ |

1. BI- Business Intelligence) a centralized data warehouse of the Ministry of Health(: BI Portal on Birth Rates and population analysis (Hebrew) published in accordance with the Ministry of Health's public information procedures from July 2023.The information displayed on the BI portal is limited to use for the internal needs of the office. Accessed on March 13, 2025
2. The Israel Democracy Institute (IDI) <https://www.idi.org.il/haredi/2023/?chapter=51973>

Malach G, Cahaner L. Annual Statistical Report on Ultra-Orthodox (Haredi) Society in Israel 2024 <https://en.idi.org.il/media/27532/idi-annual-statistical-report-on-haredi-society-2024.pdf>

1. Israel Central Bureau of Statistics Demographic Characteristics Face of Society in Israel No. 11. Gaps between the center and the periphery (Hebrew), 2019.

<https://www.cbs.gov.il/he/publications/doclib/2019/rep_11/part01_h.pdf> Accessed March 13, 2025

1. Israel Ministry of Health, Information Department, Division of Medical Technology, Information and Research. Births and infant deaths in Israel 2000-2022 (Hebrew), 2023.
2. Grisaru-Granovskya S, Gordonc ES, Haklaic Z, Samueloffa A, Schimmel MM. Effect of interpregnancy interval on adverse perinatal outcomes — a national study. Contraception. 2009 80:512–518.
3. Israel Central Bureau of Statistics. Live births in Israel by birth weight and gestational age, for the years 2000–2022 (Hebrew) [https://www.cbs.gov.il/he/publications/LochutTlushim/2019](https://www.cbs.gov.il/he/publications/LochutTlushim/2019/%D7%9C%D7%99%D7%93%D7%95%D7%AA%20%D7%97%D7%99%20%D7%9C%D7%A4%D7%99%20%D7%9E%D7%A9%D7%A7%D7%9C%20%D7%95%D7%92%D7%99%D7%9C.docx)

Explanations:

**Maternal age** – Data from the Business Intelligence (BI) portal which is a centralized data warehouse of the Ministry of Health shows age of the mother at birth for all births in Israel. The distribution among the age groups of the mothers in our study compare similarly to those in the general population for the selected years of this study.

For **population group**, the figures published by the Israel Democracy Institute (IDI) show that in 2021 and 2022 the percentage of the population for Jews and others was 66%, ultra-Orthodox made up 13% and Arabs were 21%. In the study population our percentages are slightly different with 57% religious and secular, 26% ultra-orthodox and 17% Arabs. How the population groups are defined may differ whereby the data for this study was based on a coding method for statistical geographical regions used for census data. The IDI based their data on the Israel Central Bureau of Statistics’ Social Survey results. The survey samples persons aged 20 and over who belong to the permanent population of the State of Israel. The survey is a key source for investigating the characteristics of the population according to the level of religiosity. Respondents are asked to define their level of religiosity subjectively, out of defined options. Jewish respondents were presented with these five options: Ultra-Orthodox; Religious; Traditional-religious; Traditional not-so-religious; and Not religious, secular. Non-Jewish respondents are presented with four options: Very religious; Religious; Not-so-religious; and Not religious. https://www.cbs.gov.il/en/subjects/Pages/Social-Survey.aspx

For **residence**, the distribution of the general population in 2017 reported by the Israel Central Bureau of Statistics was very similar to the figures indicated in our study population urban: 59.5/54.9 suburban: 20.7/30.0 and rural: 19.7/15.1.

In our study population 29.9% of women were **primaparas** in 2021 and 29.2% of all women giving birth in Israel were primaparas in the same year as published in the Live Births - Main Indicators, 2002-2023 of the Central Bureau of Statistics. The percentages for the other years varied slightly compared to the general population.

Regarding **intervals between pregnancies**, a national study investigating the affect of interpregnancy interval on adverse perinatal outcomes published in 2009 showed similar percentages for larger intervals but not for the very small percentage in our study for pregnancies less than a year apart. Primaparas were excluded from this study which could explain the large difference in the group showing less than one year between pregnancies.

**Birthweights** were distributed almost identically in the study population as compared to the general population cited by the Israeli Central Bureau of Statistics.

Distribution for **Gestational age** for the study population was almost identical to the general population when groupings were prepared for comparison. The researchers chose to divide the gestational age groupings according to aspects of breastfeeding which are different to those appearing in other publications.

The rate for birth of **twins** in the general population stood at 3.6 in 2022– compared to 3.5 in the study population and in 2019 the rate of 4.0 was identical.
