# Supplementary material for: Breastfeeding rates in Israel and their health policy implications
Source: Isr J Health Policy Res. 2025 May 13;14:28. doi: 10.1186/s13584-025-00689-1 (PMC12077002; doi:10.1186/s13584-025-00689-1)
Supplement: Supplementary file 8 — Supplementary material 8 [file 13584_2025_689_MOESM8_ESM.docx]

Supplementary Material

Appendix 2

To address the issue of potential **bias in self-reported data**, mothers visit the MCH clinics to monitor their infant’s growth and development, to receive guidance on various issues and to receive vaccinations based on the national childhood vaccination schedule. This preventive care for young children (from birth to six years) is provided as a designated service via Maternal Child Health Clinics (MCHC) of which there are currently some 1000 community-based MCHC. Data from the National Quality Indicators Program show a high rate of regular utilization of early MCHC services, as 91% of children had three measurements of head circumference in the first eight months of life. Upon every visit to the MCH clinic the nurse enquires about the infant’s nutrition asking the mother if the baby is breastfeeding and if the baby receives supplements. Breastfeeding status is calculated on the reports from the last visit. **Recall bias** is reduced as the visits are quite frequent in the first 24 months. The Quality Assurance Division at the Ministry of Health heads a national program for continuous monitoring of quality-of-care indicators including visits to the MCH clinics. In 2023, 84 -90% of all mothers in Israel visited the MCH clinic within three months after the birth. The percentages are presented according to the health service providers. The public health nurses are trained specifically to support mothers in the care of their young infants without judgement, therefore, bias on self-reporting on breastfeeding status is reduced.

The Ministry of Health's data world - Quality in the healthcare system (Hebrew)

https://datadashboard.health.gov.il/portal/dashboard/serviceQuality Accessed March 17, 2025.


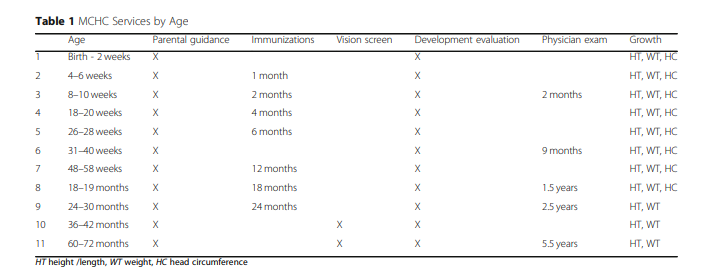


Preventive health services for young children in Israel: historical development and current challenges

Deena R. Zimmerman^1*^ [,](http://orcid.org/0000-0002-7698-3408) Gina Verbov^1^, Naomi Edelstein^1^ and Chen Stein-Zamir^1,2^ Israel Journal of Health Policy Research (2019) 9:23
