# Supplementary material for: Breastfeeding rates in Israel and their health policy implications
Source: Isr J Health Policy Res. 2025 May 13;14:28. doi: 10.1186/s13584-025-00689-1 (PMC12077002; doi:10.1186/s13584-025-00689-1)
Supplement: Supplementary file 9 — Supplementary material 9 [file 13584_2025_689_MOESM9_ESM.docx]

Supplementary Material

Appendix 3

Structured international comparison on methodologies used in similar studies from Germany, Canada, the United Kingdom, and the United States.

| Country | Type of study | Source of data | Addressing bias |
| --- | --- | --- | --- |
| Germany | Cross-sectional survey in hospitals was combined with a subsequent prospective  survey of breast-feeding and infant nutrition during the first year of life (0·5, 2, 4, 6 and 12 months after birth) | Two nationwide surveys:  Written questionnaires and phone calls  177 hospitals and 1717 mother–infant pairs  web-based questionnaires 109 hospitals and 962 mother–infant pairs | The prospective assessment minimised recall bias.  The online approach enabled mothers to answer  questionnaires within 15 min, bypassing the postal  mail and eliminating the risk of interviewer bias  during the phone interviews  The hospital sample in the SuSe II study  comprised relatively more baby-friendly hospitals  Therefore, the risk of a selection bias towards an overestimation  of the breast-feeding friendliness in  Germany cannot be excluded. |
| Canada | National cross-sectional  Canadian Community Health Survey. | This survey provides  the most current and nationally representative data on  breastfeeding, with a response rate of 60.7%. 5,392 females aged 15–55 who had given  birth in the five years preceding the survey  The  remaining 4,845 females (weighted to 1,471,316 females  representative of the Canadian population) constituted  the cohort used for the “any breastfeeding” analysis. | Due to the self-reported nature of the survey,  reports of breastfeeding experiences may be subject  to recall bias and social desirability bias. |
| United Kingdom | Two national maternity cross-sectional postal and online surveys six months after the birth. | Random population-based samples of approximately 16,000 women were identified by the Office for National Statistics (ONS) using birth registration records | Response rate to the surveys was 28% in 2018 (n = 4,509) and 29% in 2020  Corrections for these differences applied survey weights derived  using maternal age, marital/registration status, whether born outside the UK, region of residence,  area deprivation (measured by the Index of Multiple Deprivation, IMD), and parity. |
| United States | Pregnancy Risk Assessment and Monitoring System (PRAMS) Survey data from 2016 to 2017 asking new mothers questions about their experiences and behaviors before and during pregnancy, right after delivery, and in the early infancy period. | N = 20,694  The PRAMS sample is randomly drawn from birth certificates in participating states. Mothers are selected for participation between 2-6 months after delivery, with the majority chosen 2 months after delivery. Selected women are first contacted by mail. If there is no response to repeated mailings, women are contacted and given the opportunity to answer the survey by telephone. | Study variables were summarized by using weighted means and proportions and compared according to parity by using Wald tests. |
